# Brazilian Red Propolis Induces Apoptosis-Like Cell Death and Decreases Migration Potential in Bladder Cancer Cells

**DOI:** 10.1155/2014/639856

**Published:** 2014-11-03

**Authors:** Karine Rech Begnini, Priscila Marques Moura de Leon, Helena Thurow, Eduarda Schultze, Vinicius Farias Campos, Fernanda Martins Rodrigues, Sibele Borsuk, Odir Antônio Dellagostin, Lucielli Savegnago, Mariana Roesch-Ely, Sidnei Moura, Francine F. Padilha, Tiago Collares, João Antonio Pêgas Henriques, Fabiana Kömmling Seixas

**Affiliations:** ^1^Programa de Pós-Graduação em Biotecnologia (PPGB), Biotecnologia/Centro de Desenvolvimento Tecnológico, Universidade Federal de Pelotas, Campus Universitário s/n, 96010-900 Capão do Leão, RS, Brazil; ^2^Grupo de Pesquisa em Oncologia Celular e Molecular (GPO), Biotecnologia/Centro de Desenvolvimento Tecnológico, Universidade Federal de Pelotas, Campus Universitário s/n, 96010-900 Capão do Leão, RS, Brazil; ^3^Universidade de Caxias do Sul, Caxias do Sul, RS, Brazil; ^4^Universidade Tiradentes, Aracaju, SE, Brazil

## Abstract

Natural products continue to be an invaluable resource of anticancer drug discovery in recent years. Propolis is known for its biological activities such as antimicrobial and antitumor effects. This study assessed the effects of Brazilian red propolis (BRP) on apoptosis and migration potential in human bladder cancer cells. The effect of BRP ethanolic extract (25, 50, and 100 *μ*g/mL) on 5637 cells was determined by MTT, LIVE/DEAD, and migration (scratch assay) assays. Apoptosis induction was investigated through flow cytometry and gene expression profile was investigated by qRT-PCR. Results showed cytotoxicity on MTT and LIVE/DEAD assays, with IC_50_ values of 95 *μ*g/mL in 24 h of treatment. Cellular migration of 5637 cells was significantly inhibited through lower doses of BRP ethanolic extract (25 and 50 *μ*g/mL). Flow cytometry analyses showed that BRP induced cytotoxicity through apoptosis-like mechanisms in 5637 cells and qRT-PCR revealed increased levels of Bax/Bcl-2 ratio, p53, AIF, and antioxidant enzymes genes. Data suggest that BRP may be a potential source of drugs to bladder cancer treatment.

## 1. Introduction

Cancer is one of the leading causes of death in both developing and developed countries and is a worldwide concern. A total of 1,660,290 new cancer cases and 580,350 cancer deaths are projected to occur in the United States in 2013 [1] and by 2050, 27 million new cancer cases and 17.5 million cancer deaths are projected to occur in the world [2]. An analysis of the anticancer drugs revealed that 47.1% of the approved anticancer drugs were either unmodified natural products or their semisynthetic derivatives or synthesized molecules based on natural product compound pharmacophores [3]. Natural products tend to present more structurally diverse “drug-like” and “biologically friendly” molecular qualities than pure synthetic compounds at random [4] and have been considered as an “unlimited” resource for future drug discovery [5].

Propolis is a resinous mixture of substances collected by honey bees (*Apis mellifera*) from various plant sources. It has been used in folk medicine for centuries mostly due to its antimicrobial and anti-inflammatory activities [6]. Notable chemical differences are often found between propolis samples and Brazil has the widest chemical diversity of propolis types [7]. Brazilian red propolis (BRP) is the newest variety of Brazilian propolis and is a promising source of new bioactive compounds [8] like chalcones, pterocarpans, isoflavonoids, and polyphenols [9].

Since its discovery, BRP has been studied to elucidate its several biological properties. Studies have shown antitumor properties of red propolis against several types of cancer both* in vitro* and* in vivo* [8, 10–14]. The mechanisms involved on potential anticancer effects of propolis are suppressing cancer/precancerous cells proliferation via its immunomodulatory effect; decreasing the cancer stem cell populations; blocking specific oncogene signaling pathways; modulating the tumor microenvironment; and, lastly, being an adjunct or complementary treatment to existing mainstream anticancer therapies [15]. Besides that, BRP had also shown a potent antiangiogenic activity by targeting key steps that are required for new blood vessel development [16, 17], showing a natural chemopreventive activity.

The aim of this study was to investigate whether Brazilian red propolis ethanolic extracts have cytotoxic effect and study the underlying cell death mechanisms in human bladder cancer cells.

## 2. Materials and Methods

### 2.1. Red Propolis Sample and Extract Preparation

The red propolis was collected from a geographic region on northeast of Brazil known as Brejo Grande (S 10°28′25′′ and W 36°26′12′′). The samples of red propolis were collected in September 2011 and frozen at −20°C. For extract preparation, 1 g (dry weight) of raw red propolis was mixed with 10 mL of EtOH-H_2_O 70% (v/v) and shaken at room temperature for 24 h. After extraction, the mixture was filtered and the solvent was evaporated and produced a red fine powder. This dry extract was kept frozen at −20°C. The BRP final concentrations (25, 50, and 100 *μ*g/mL) were prepared immediately before use with EtOH-H_2_O 50% (v/v).

### 2.2. Chemical Characterization of Red Propolis Extract (Mass Analysis)

The dries extracts were dissolved in a solution of 50% (v/v) chromatographic grade acetonitrile (Tedia, Fairfield, OH, USA), 50% (v/v) deionized water, and 0.1% formic acid. The solutions were infused directly individually into the ESI source by means of a syringe pump (Harvard Apparatus) at a flow rate of 50 *μ*L min^−1^. ESI(+)-MS and tandem ESI(+)-MS/MS were acquired using a hybrid high-resolution and high accuracy (5 *μ*g/L) microTof (Q-TOF) mass spectrometer (Bruker Scientific) under the following conditions: capillary and cone voltages were set to +3500 V and +40 V, respectively, with a desolvation temperature of 100°C. For ESI(+)-MS/MS, the energy for the collision induced dissociations (CID) was optimized for each component. Diagnostic ions in different fractions were identified by the comparison of their ESI(+)-MS/MS dissociation patterns with compounds identified in previous studies. For data acquisition and processing, compass software (Bruker Scientific) was used. The data were collected in the* m/z* range of 70–800 at the speed of two scans per second, providing the resolution of 50,000 (FWHM) at* m/z* 200. No important ions were observed below* m/z* 180 or above* m/z* 650; therefore ESI(+)-MS data is shown in the* m/z* 180−650 range.

### 2.3. Cell Culture

The human bladder carcinoma cell line (5637) was obtained from the Rio de Janeiro Cell Bank (PABCAM, Federal University of Rio de Janeiro, RJ, Brazil) and cultured as a monolayer in Dulbecco's modified Eagle's medium (DMEM) (Vitrocell Embriolife, Campinas, Brazil), supplemented with 10% fetal bovine serum (FBS) (Gibco, Grand Island, NY, USA), 1% L-glutamine, and 1% penicillin/streptomycin. Cells were grown at 37°C in an atmosphere of 95% humidified air and 5% CO_2_.

### 2.4. Antiproliferative Assay

The proliferation of the 5637-cell line after treatment was determined by measuring the reduction of soluble MTT to water insoluble formazan. Cells were seeded at a density of 2 × 10^4^ cell per well in a volume of 100 *μ*L in 96-well plates and grown at 37°C in a 5% CO_2_ atmosphere for 24 h before being used in the cell viability assay. Cells were then treated with the red propolis extract EtOH-H_2_O 50% (v/v) at concentrations of 25, 50, and 100 *μ*g/mL or EtOH-H_2_O 50% vehicle alone, for 24 h. Following incubation, 20 *μ*L of MTT was added to each well, and the cells were incubated for an additional 3 hours at 37°C. Differences in total cellular metabolism were detected at a wavelength of 492 nm using a microplater reader. The inhibition (%) of cell proliferation was determined as follows: inhibitory growth = (1 − Abs492_treated  cells_/Abs492_control  cells_) × 100% [18]. The IC_50_ (concentration *μ*g/mL that inhibits 50% of cell growth) was also calculated using GraphPad Prism 5.0 Software. The normal CHO-K1 cell line was used as selectivity control in this test. All observations were validated by at least three independent experiments in triplicate for each experiment.

### 2.5. LIVE/DEAD Assay

Cells were treated with red propolis extract EtOH-H_2_O 50% (v/v) at concentrations 25, 50, and 100 *μ*g/mL for 24 h as described above. LIVE/DEAD cell viability assay (Invitrogen, Carlsbad, CA, USA) was conducted following the manufacturer's instructions. Live cells were able to take up calcein and could be analyzed by green fluorescent light emission (488 nm). Ethidium bromide homodimer diffuses through the now permeable membrane of dead cells and binds to DNA, which was detected by the red fluorescent signal (546 nm). The LIVE/DEAD assay was analyzed with a fluorescence microscope Olympus IX71 (Olympus Optical Co., Tokyo, Japan) by multicolour imaging. After excitation at 480 nm and emission at 510 nm the fluorescent images were stored as TIFF files using a digital camera attached to a fluorescence microscope (DP 12; BX 51; Olympus, Tokyo, Japan). The recorded images were analyzed using Cell^∧^F software (Cell-F, New York, USA). The data were expressed as the mean ± SEM and the experiment was run in triplicate.

### 2.6. Apoptosis Assays

Apoptosis was determined by flow cytometry using Annexin V-7AAD apoptosis detection kit (Guava Technologies, Millipore Corporation) and TUNEL detection kit (Guava Technologies, Millipore Corporation), following the manufacturer's instructions. 5637 cells were exposed to red propolis extract EtOH-H_2_O 50% (v/v) at concentrations of 25, 50, and 100 *μ*g/mL for 24 h in culture media at 37°C with 5% CO_2_. A range of 2.0 × 10^4^ to 1.0 × 10^5^ treated cells (100 *μ*L) were added to 100 *μ*L of Guava Nexin reagent. Cells were incubated in the dark at room temperature for 20 min and samples were acquired on the flow cytometry (Guava Flow Cytometry easyCyte System; Millipore Corporation). In this assay, an Annexin V-negative and 7-AAD-positive result indicated nuclear debris; an Annexin V-positive and 7-AAD-positive result indicated late apoptotic/death cells, while an Annexin V-negative and 7-AAD-negative result indicated live healthy cells and Annexin V-positive and 7-AAD-negative result indicated the presence of early apoptotic cells. The results were reported as the percentage of cells in each apoptotic phase (early and late) and the normal CHO-K1 cell line was used as selectivity control in this test.

For TUNEL assay 5637 cells were subjected to cells fixation procedure with 50 *μ*L of 4% (w/v) paraformaldehyde in PBS for 60 min at 4°C and then with 200 *μ*L of ice-cold 70% (v/v) ethanol at −20°C for at least 18 h. For staining procedure, 1.5 × 10^4^ to 1.0 × 10^5^ of fixed cells was washed twice and was added to 25 *μ*L of DNA Labeling Mix for 60 minutes at 37°C. At the end of the incubation time, cells were centrifuged and suspended in 50 *μ*L of the Anti-BrdU Staining Mix. Cells were incubated in the dark at room temperature for 30 min and samples were acquired on the flow cytometry (Guava Flow Cytometry easyCyte System; Millipore Corporation). In this assay, terminal deoxynucleotidyl transferase (TdT) catalyzes the incorporation of BrdU residues into the fragmenting nuclear DNA of apoptotic cells at the 3′-hydroxyl ends by nicked-end labeling. TRITC-conjugated anti-BrdU antibody binds to the incorporated BrdU residues, labeling the mid- to late-stage apoptotic cells.

### 2.7. Quantitative Real-Time PCR (qRT-PCR)

The gene expression profiles of apoptotic and oxidative stress-related genes were investigated by qRT-PCR. Cells were added to 6-well flat bottom plates at a density of 2 × 10^5^ per well and grown at 37°C in a humidified atmosphere of 5% CO_2_, 95% air for 24 h. The cells were then treated with the red propolis extract EtOH-H_2_O 50% (v/v) at concentrations of 25, 50, and 100 *μ*g/mL for 24 h. Total RNA extraction, cDNA synthesis, and qRT-PCR were conducted as previously described [19]. Briefly, RNA samples were isolated using TRIzol reagent (Invitrogen, USA) and samples were DNase-treated with a DNA-free kit (Ambion, USA) following the manufacturer's protocol. First-strand cDNA synthesis was performed with 700 ng of RNA using the High Capacity cDNA Reverse Transcription Kit (Applied Biosystems, UK) according to the manufacturer's protocol. Real-time PCR reactions were run on a Stratagene Mx3005P Real-Time PCR System (Agilent Technologies, USA) using SYBR Green PCR Master Mix (Applied Biosystems, UK) and the primers described in Table 1.

### 2.8. Migration Assay

The ability of cells to migrate in monolayer cultures was assessed by a scratch-wound assay [20]. Confluent 5637-cell cultures in 6-well flat bottom plates were scraped with a p200 pipet tip to create a wide cell-free zone with a straight wound edge. Cells were grown in media with 25 and 50 *μ*g/mL of red propolis EtOH-H_2_O extract for 24 h. The edge of the wound was marked at the bottom of the plate with a fine gauge hypodermic needle as a migration reference point. The distance and quantity of cell migration into the cell-free zone were evaluated on a digital camera attached to an inverted microscope for 12 h (DP 12; BX 51; Olympus, Tokyo, Japan). The recorded images were analysed using Cell^∧^F software (Cell-F, New York, USA). The data were expressed as the mean ± SEM and the experiment was run in triplicate.

### 2.9. Data Analysis

Data sets were analyzed using one-way or two-way ANOVA followed by a Tukey test for multiple comparisons, except for Bax/Bcl-2 ratio that was analyzed using Student *t*-test. Significance was considered at *P* < 0.05 in all analyses. Data were expressed as mean ± SEM.

## 3. Results

### 3.1. Chemical Characterization of Red Propolis Extract (Mass Analysis)

**Figure 1:**
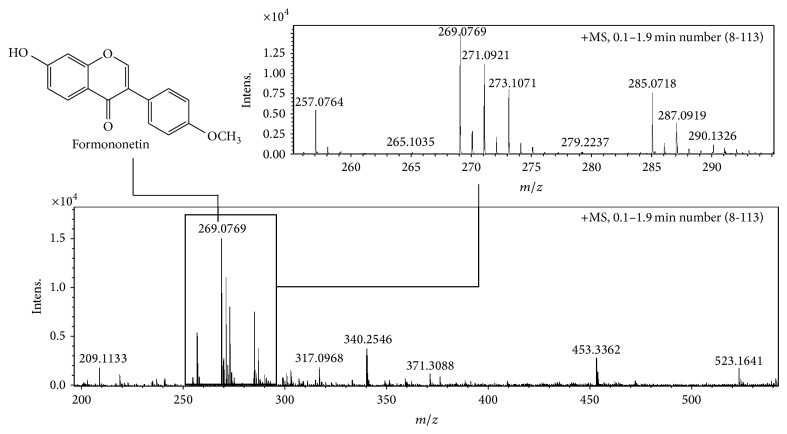
ESI(+)-MS fingerprint of red propolis ethanolic extract.

Due to environmental conditions, the chemical composition of propolis extracts may differ. As reported in a previous work, high-resolution direct-infusion mass spectrometry (HR-DIMS) was used for chemical characterization of the red propolis extract [14]. The main components were maintained as follows:* m/z* 257.0764 (liquiritigenin); 269.0769 (formononetin); 271.0921 (medicarpin); 285.0718 (biochanin A); 523.1641 (retusapurpurin B) (Figure 1). Exact mass, fragmentation pathway, and isotopic ratio were used for confirmation.

### 3.2. Red Propolis Inhibited Cell Proliferation and Increased 5637-Cell Death

**Figure 2:**
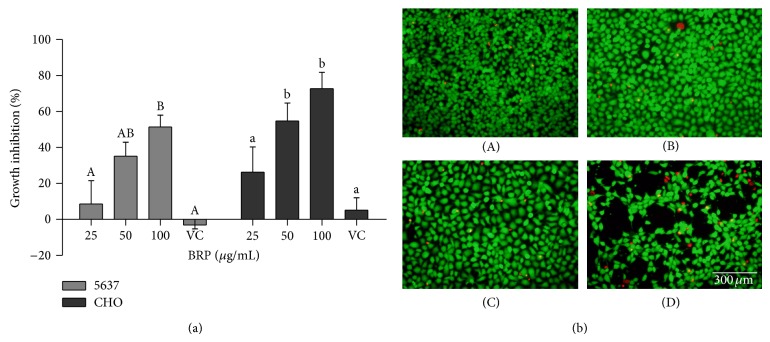
Brazilian red propolis ethanolic extract increased antiproliferative and cytotoxicity effect in 5637 cells (a). Cell proliferation in 5637 and CHO-K1 was investigated by MTT assay. Data are expressed as means ± SEM from three independent experiments. Different letters (A/a, B/b, and C/c) indicate significant differences between the means. Uppercase letter indicates difference between the treatments in 5637 cells. Lowercase letter indicates difference between the treatments in CHO-K1 cells. The differences were considered significant at *P* < 0.05. VC = vehicle control (EtOH-H_2_O). (b) Brazilian red propolis ethanolic extract increased 5637-cell death. Bladder cancer cells were treated with the BRP extract for 24 h. Analysis of cell death was estimated by LIVE/DEAD assay with 20x optical zoom. Untreated cells (A); 25 *μ*g/mL treatment (B); 50 *μ*g/mL treatment (C); 100 *μ*g/mL treatment (D).

The result showed that red propolis extract significantly decreased 5637-cell viability* in vitro* in a dose-dependent manner (Figure 2(a)). The cell growth inhibition following red propolis treatment was over 50% from 100 *μ*g/mL in 24 hours. EtOH-H_2_O vehicle alone showed no cytotoxicity or antiproliferative activity at 24 h of treatment. The* in vitro *cytotoxic activity of red propolis extract showed an IC_50_ value of 95 *μ*g/mL in 24 h of treatment. BRP treatment also inhibited proliferation in CHO-K1 cell line at 24 h of treatment, displaying no selectivity between normal and cancer cells in terms of* in vitro* growth inhibition (Figure 2(a)).

LIVE/DEAD assay showed an increase in cell death (red fluorescence) after red propolis treatments compared to the control group (Figure 2(b)). Additionally, a reduction in cell number can clearly be observed at concentrations of 100 *μ*g/mL (Figure 2(b)(D)). EtOH-H_2_O vehicle alone treatment promoted cell death similar to that observed in the control group (data not shown).

### 3.3. Red Propolis Induced Apoptosis on 5637 Cells

**Figure 3:**
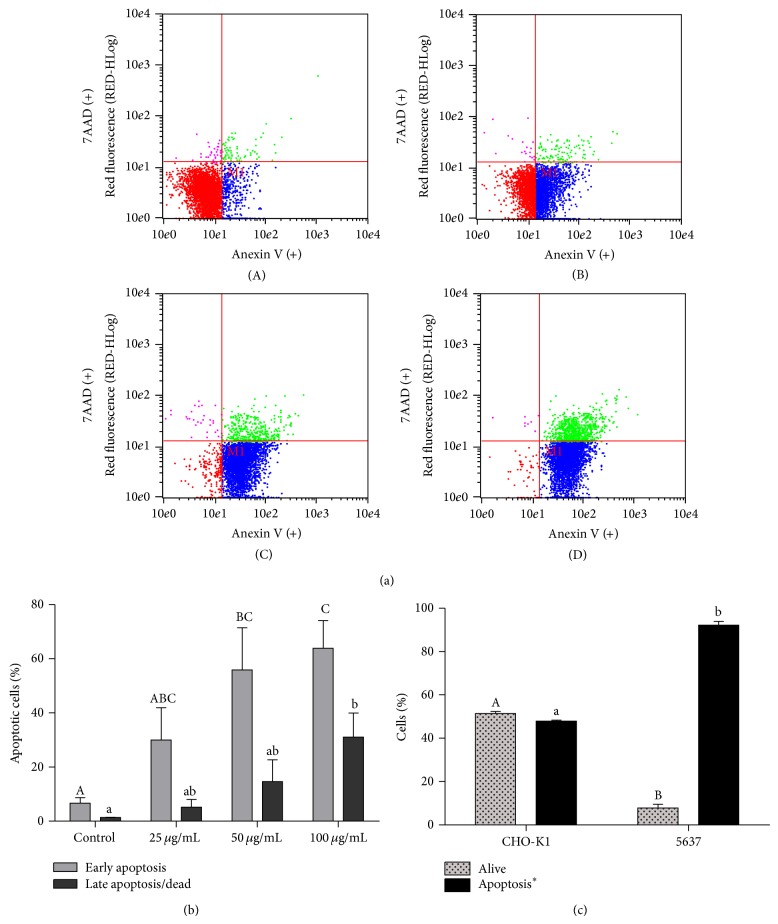
BRP ethanolic extract promotes apoptosis in 5637 cells. (a) Flow cytometry graphs for Annexin V-7AAD analysis of 5637 cells treated with 25, 50, and 100 *μ*g/mL of BRP ethanolic extract for 24 h. Control group (A), 25 *μ*g/mL group (B), 50 *μ*g/mL group (C), and 100 *μ*g/mL group (D). (b) Percentage of apoptotic cells after treatment with 25, 50, and 100 *μ*g/mL of BRP ethanolic extract for 24 h. Data are expressed as means ± SEM from three independent experiments. Different letters (A, B, and C) indicate significant differences between the means and differences were considered significant at *P* < 0.05. (c) Selectivity of BFP in normal and tumoral cells after treatment with 100 *μ*g/mL for 24 h. Data are expressed as means ± SEM from three independent experiments. Different letters (A, B, and C) indicate significant differences between the means and differences were considered significant at *P* < 0.05. (∗) Early and late apoptosis together.

The results indicated that red propolis is capable of inducing early apoptosis at concentrations of 50 and 100 *μ*g/mL (55.8% and 63.9%, resp.) when compared to the control group (*P* < 0.05) (Figure 3); however no apoptosis difference is observed between these two concentrations (*P* > 0.05). The concentration of 25 *μ*g/mL was not effective (*P* > 0.05) in inducing early apoptosis, presenting levels of apoptosis similar to untreated control cells (29.9 and 6.7%, resp.). The red propolis extract induced a higher percentage of late apoptosis/dead at 100 *μ*g/mL concentration (31.1%) compared to the control (*P* < 0.05). At the 25 and 50 *μ*g/mL concentrations the percentage of late apoptotic/dead cells was 5.1% and 14.6%, respectively, similar to that observed in the untreated control group (*P* > 0.05). Exposure of the 5637 cells to EtOH-H_2_O vehicle alone had no effect in apoptosis induction (data not shown). BRP treatment was not able to induce apoptosis in CHO-K1 cell line at 25 and 50 *μ*g/mL. The percentage of apoptotic cells was 11% and 15.4% for 25 and 50 *μ*g/mL, respectively, which is not different from untreated control group (*P* > 0.05) (data not shown). Early and late apoptotic levels together at 100 *μ*g/mL were 47% in CHO-K1 cells. Interestingly, 51% of CHO-K1 normal cells remained alive after 100 *μ*g/mL BRP treatment but only 8% of 5637 tumoral cells remained alive after the same treatment. This result may indicate that BRP displays selectivity between normal and cancer cells in terms of* in vitro* apoptosis induction (Figure 3(b)).

**Figure 4:**
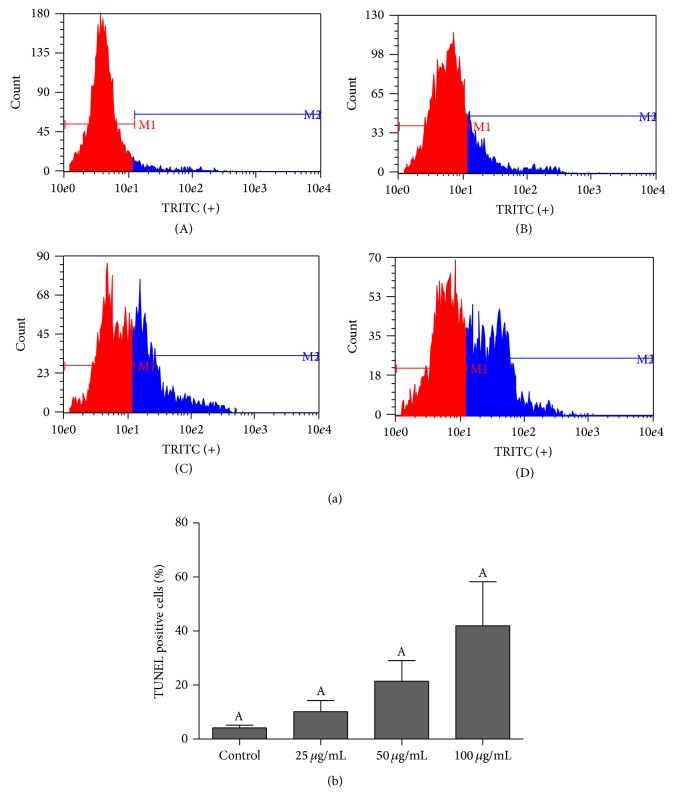
BRP ethanolic extract increased DNA fragmentation in 5637 cells. (a) Flow cytometry graphs for TUNEL analysis of cells treated with 25, 50, 100, and 200 *μ*g/mL of BRP ethanolic extract for 24 h. Control group (A), 25 *μ*g/mL group (B), 50 *μ*g/mL group (C), and 100 *μ*g/mL group (D). (b) Percentage of cells with DNA fragmentation after treatment with 25, 50, and 100 *μ*g/mL of BRP ethanolic extract for 24 h. Data are expressed as means ± SEM from three independent experiments. Different letters (A and B) indicate significant differences between the means and differences were considered significant at *P* < 0.05.

TUNEL staining assay was performed to better elucidate the mid- to late-stage apoptosis of 5637 cells induced by red propolis, once that 7AAD staining does not differentiate between late-stage apoptoses from another cell death types.  Figure 4 demonstrates that red propolis presented a tendency to increase late apoptosis inducement; however no differences (*P* > 0.05) on late apoptosis rates were observed between control group and red propolis treatment at 25, 50, and 100 *μ*g/mL in bladder cancer cells (Figure 4(b)).

### 3.4. Red Propolis Changes Apoptotic Gene Expression Profile on 5637 Cells

**Figure 5:**
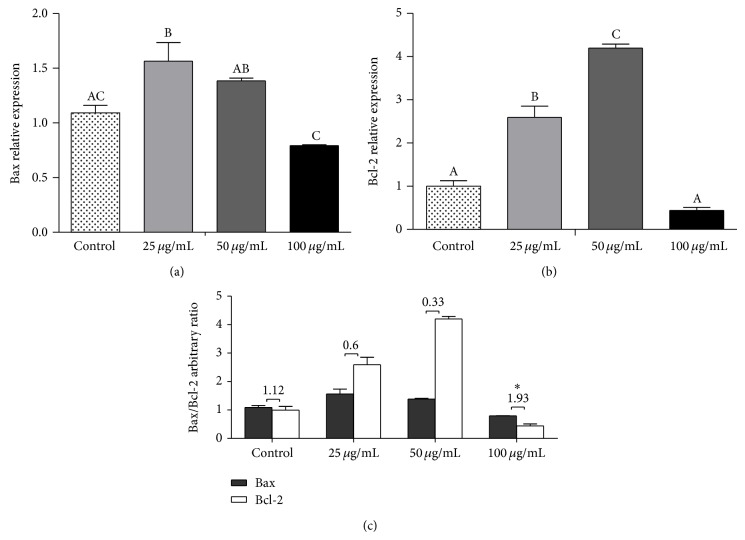
BRP ethanolic extract increased the Bax/Bcl-2 ratio in 5637-cell line after 24 hours of treatment. The gene expression profile was determined by qRT-PCR and data were normalized using GAPDH levels. (a) Proapoptotic Bax gene expression. (b) Antiapoptotic Bcl-2 gene expression. (c) Bax/Bcl-2 ratio. Data are expressed as means ± SEM from three independent experiments. Different letters (A, B, and C) indicate significant differences between the means and (∗) indicates difference between 100 *μ*g/mL treated group and no treatment control group. The differences were considered significant at *P* < 0.05.

The expression levels of pro- and antiapoptotic genes (Bax, Bcl-2, AIF, Endo G, caspase-3, caspase-8, caspase-9, and p53) in 5637 cells were evaluated by qRT-PCR. As shown in Figure 4, the expression of Bax was bell-shaped, with 25 *μ*g/mL treatment showing the higher fold induction (Figure 5(a)). However, Bcl-2 in 5637 cells was also increased after 25 and 50 *μ*g/mL treatments when compared to the control (*P* < 0.05), showing 1.5- and 2-fold increase in mRNA expression, respectively (Figure 5(b)). Interestingly, no effect in mRNA expression levels was observed after 100 *μ*g/mL treatment in 5637 cells when compared to the control (*P* > 0.05), showing that red propolis extract may trigger a negative feedback. However, the Bax/Bcl-2 ratio increased in 5637 cells after 100 *μ*g/mL BRP treatment (Figure 5(c)), compared to that observed in untreated cells and 25 and 50 *μ*g/mL treated cells (*P* < 0.05).

**Figure 6:**
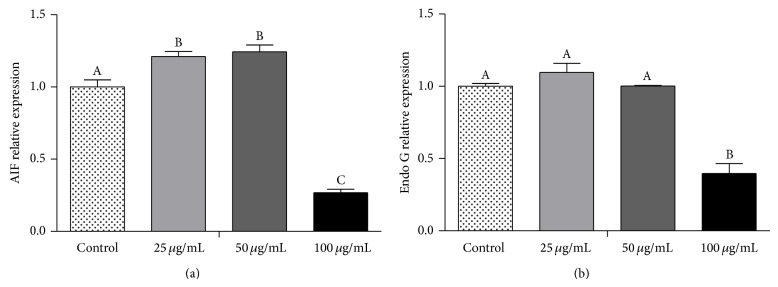
Effect of BRP ethanolic extract in apoptotic-related gene expression of 5637-cell line. (a) AIF; (b) Endo G. Data are expressed as means ± SEM from three independent experiments. Different letters (A, B, and C) indicate significant differences between the means and differences were considered significant at *P* < 0.05.

Apoptosis-inducing factor (AIF) mRNA expression was found to be significantly upregulated in 25 and 50 *μ*g/mL red propolis treated cells (*P* < 0.05) compared to no treated control cells and to 100 *μ*g/mL treated cells (Figure 6(a)). No differences were observed between the controls 25 and 50 *μ*g/mL of red propolis treatments in Endo G gene expression (Figure 6(b)). However, both Endo G and AIF mRNA levels were significantly lower (*P* < 0.05) in the 100 *μ*g/mL treatment compared to the control.

**Figure 7:**
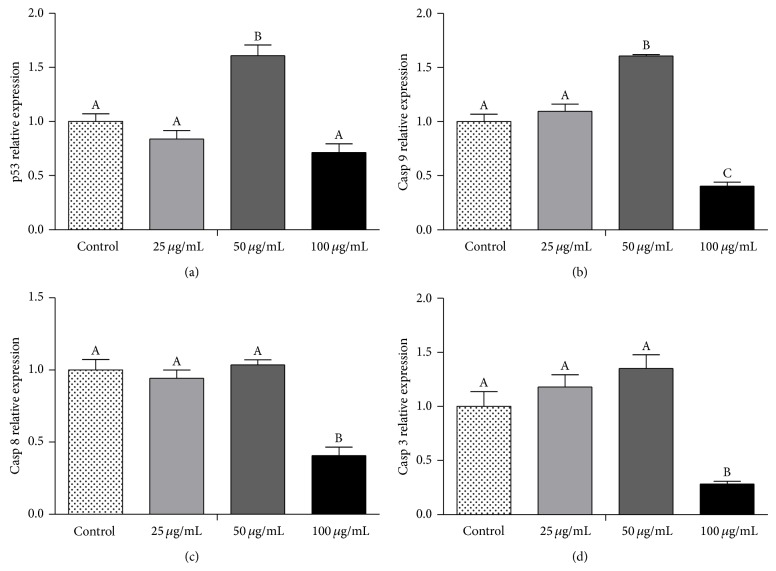
BRP ethanolic extract induced changes in the mRNA levels in 5637 cells. 5637 cells were treated with the indicated concentrations of BRP ethanol extract for 24 h. RT-PCR were performed to caspase-9, caspase-8, caspase-3, and p53 genes and data were normalized using GAPDH levels. Different letters (A, B, and C) indicate significant differences between the means and differences were considered significant at *P* < 0.05.

Red propolis also induced changes in the mRNA levels of caspase-9, caspase-8, caspase-3, and p53 in 5637 cells (Figure 7). Initiator caspase-9 and p53 gene were significantly upregulated (*P* < 0.05) by 50 *μ*g/mL treatment compared to control cells and other concentrations treatments (Figures 7(a) and 7(b)). Additionally, caspase-3, caspase-8, and caspase-9 were downregulated by 100 *μ*g/mL treatment compared to no treated and to 25 and 50 *μ*g/mL treated cells (*P* < 0.05) (Figures 7(b)–7(d)).

### 3.5. Red Propolis Increased Gene Expression of Antioxidant Enzymes

**Figure 8:**
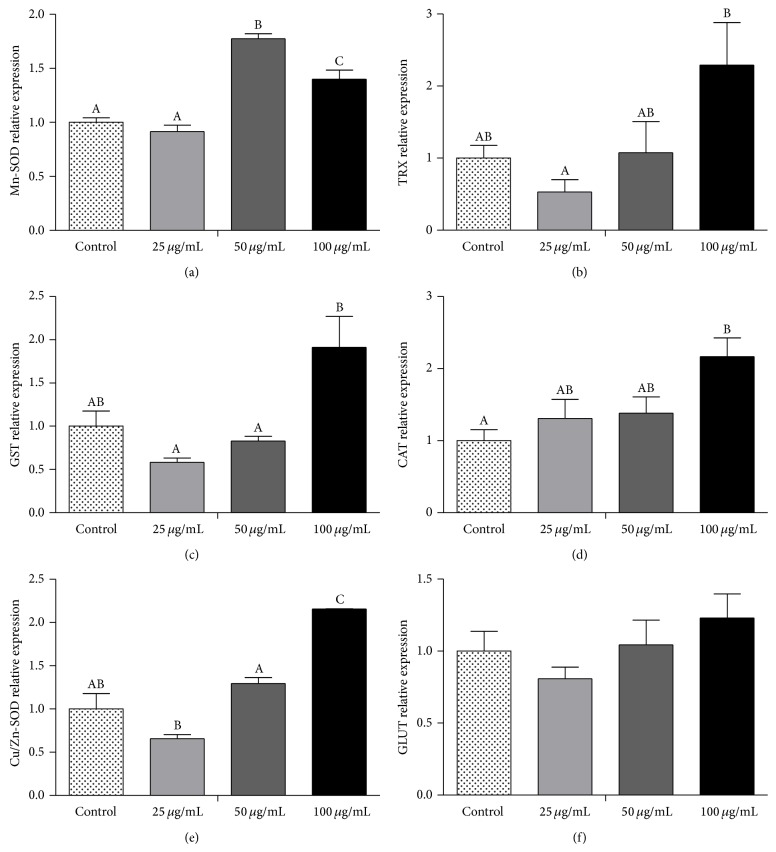
BRP ethanolic extract changes antioxidant enzymes gene expression in 5637 cells. The gene expression profile was determined by qRT-PCR. (a) Mn-SOD; (b) TRx; (c) GST; (d) CAT; (e) Cu/Zn-SOD; (f) GLUT. Data are expressed as means ± SEM from three independent experiments. Different letters (A, B, and C) indicate significant differences between the means and differences were considered significant at *P* < 0.05.

The gene expression profiles of the following enzymes were investigated in this study as follows: catalase (CAT), Cu/Zn superoxide dismutase (Cu/Zn-SOD), manganese superoxide dismutase (Mn-SOD), glutathione-S-transferase (GST), and thioredoxin reductase-1 (TRX). Red propolis showed a tendency to increased CAT, Cu/Zn-SOD, and Mn-SOD mRNA levels significantly (*P* < 0.05) in cells exposed to 50 and 100 *μ*g/mL compared to the controls (Figures 8(a), 8(d), and 8(e)); however there was no difference in CAT mRNA expression between 50 and 100 *μ*g/mL treatments (Figure 8(d)). No difference between the control and 25 *μ*g/mL treatment was observed for these genes expression levels. Moreover, the GST, TRX, and GLUT mRNA expression patterns were investigated and no differences between treated and untreated cells were observed (Figures 8(b), 8(c), and 8(f)), but treated cells presented a dose-dependent tendency to increased expression levels in TRX and GST genes.

### 3.6. Red Propolis Inhibits Migration of Urothelial Carcinoma

**Figure 9:**
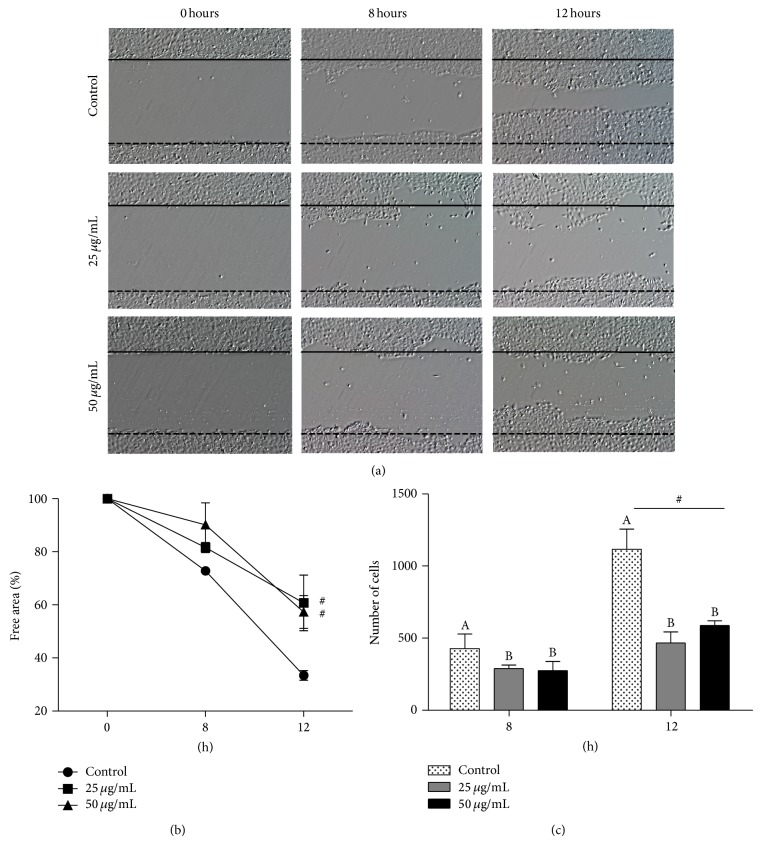
BRP ethanol extract decreased migration of 5637-cell line after 25 and 50 *μ*g/mL treatments. (a) Migration of 5637 cells after 24 hours of treatment with 25 and 50 *μ*g/mL of BRP ethanolic extract. (b) Percentage of free width in the scratch-wound healing after treatment with 25 and 50 *μ*g/mL of BRP ethanolic extract in 0, 8, and 12 h. (c) Number of cells that cross the scratch-wound healing after treatment with 25 and 50 *μ*g/mL of BRP ethanolic extract in 8 and 12 h. Data are expressed as means ± SEM from three independent experiments. Different letters (A and B) and asterisk (∗) indicate significant differences between the means and differences were considered significant at *P* < 0.05. ^#^ *P* < 0.01.

Migration of 5637 cells was significantly inhibited by 24 h of red propolis treatment. As shown in Figure 9, cellular migration was controlled in time-dependent by red propolis ethanolic extract. The width of the scratch-wound healing was inhibited by up to 20% and 30% at 8 and 24 h of incubation, respectively, in both concentrations of red propolis tested (Figures 9(a) and 9(b)). The number of cell migration to the scratch-wound healing was also smaller when cells were treated with BRP compared to those who were not treated (Figures 9(a) and 9(c)). Furthermore, inhibition of 5637 migrations occurred at lower concentrations (25 and 50 *μ*g/mL) than the aforementioned IC_50_ concentrations by MTT assay.

## 4. Discussion

Natural products continue to be an invaluable resource of anticancer drug discovery [5]. The prospect of using natural products to create more selective and effective cancer treatment is a reality and propolis and its compounds possess strong antitumor potential [15, 21]. In the present study we evaluated for the first time the effect of Brazilian red propolis ethanolic extract on bladder cancer cellular model. Our* in vitro* data demonstrated that red propolis treatment above 50 *μ*g/mL resulted in morphological changes, significant antiproliferative effect, and cytotoxic effect in bladder cancer cell line. Interestingly, our results have also shown that lower concentrations of red propolis treatment (25 and 50 *μ*g/mL) are able to significantly decrease bladder cancer cells migration* in vitro*. These data indicate a strong effectiveness of the BRP extract against bladder cancer cell line.

Apoptosis induction is one of the mechanisms proposed for the anticancer therapeutic effects of propolis [22, 23]. Apoptosis is a well-characterized type of programmed cell death (PCD) and is considered as a highly regulated process that allows a cell to self-degrade in order to eliminate an unwanted or dysfunctional cell [24]. Conventional anticancer treatments, such as chemotherapy and radiotherapy, kill tumor cells primarily by the induction of apoptosis or apoptosis-like PCD [24, 25]. In this study we demonstrate by flow cytometry that red propolis might be an important apoptosis inductor in bladder cancer cells, showing an increase in both early and late apoptosis stages* in vitro*. More than that, the mechanism of apoptosis induced by BRP seems to be dependent on the concentration of the propolis extract.

A single family of proteases, the caspases, has long been considered as the pivotal executioner of all programmed cell deaths [25]. When activated, the caspases cleave a series of substrates, activate DNAses, and orchestrate cell death [26]. However, there are evidences that apoptosis can occur independently of caspases activity [27]. Apoptosis-like PCD is a programmed cell death that shows a less compact/complete chromatin condensation than in apoptosis and most of the published forms of caspase-independent apoptosis fall into this class of PCD [25]. More than that, one of the main characteristics of PCD is the fragmentation of nuclear DNA [27]. Apoptosis-inducing factor (AIF) is a flavoprotein that resides in the mitochondrial intermembrane space [28]. Upon induction of apoptosis, AIF is translocated from the mitochondria to the nucleus and it causes chromatin condensation and large-scale DNA fragmentation without caspases activation [28–30]. Herein, ethanol extract of red propolis does not induce significant caspases expression activities. On the other hand, the AIF gene expression profile in the 5637-cell line increased after BRP treatment and an increase of DNA fragmentation was observed after 24 h of BRP treatment. The apoptosis gene expression data from our experiments confirmed the results of cytotoxicity and apoptosis assays, showing that BRP extract may induce apoptosis or apoptosis-like PCD in 5637 cells and that this may occur by activation of different apoptosis pathways.

The positive effect of propolis anticancer therapy is seen in its ability to initiate apoptosis in cancer cells through both the intrinsic and extrinsic pathway [22, 31–35]. The intrinsic apoptotic pathway is mediated by the mitochondria and is mainly controlled by the balance and interactions between pro- and antiapoptotic members of the Bcl-2 family proteins, which regulate the permeability of the mitochondrial membrane [26]. It has been proposed that the ratio between Bcl-2 and Bax genes is more important in the regulation of apoptosis than the level of each Bcl-2 family protein alone [36] and the ratio of death and survival signals sensed by the Bcl-2 family proteins determines whether the cell will live or die [26, 37, 38]. Although both Bax and Bcl-2 genes have shown an increase in expression profile after treatment with BRP in this study, the Bax/Bcl-2 ratio in the 5637-cell line strongly increased after 100 *μ*g/mL of BRP treatment, suggesting that Bax and Bcl-2 may be involved in the apoptotic events associated with the cytotoxic effects of BRP. More than that, our study also showed an increase in p53 gene expression after treatment with BRP extract. It is well known that p53 contributes to apoptosis induction mostly by its transcription-dependent effects. However, it has been shown that p53 can also induce cell death via direct activation of Bcl-2, Bcl-XL, and Bax [39–41]. These data support our speculation that Brazilian red propolis may trigger apoptosis or apoptosis-like PCD induction through p53, Bax, and Bcl-2 activation.

The established role of antioxidant enzymes against cancer is in the prevention of oxidative DNA damage and reactive oxygen species (ROS) formation [42, 43]. It has been shown that propolis has the ability to scavenge the free radicals in rats [44]. Oxidative stress can trigger endoplasmic reticulum (ER) stress [45] and ER stress is able to induce apoptosis without involvement of caspases [46]. Moreover, the regulation of ER membrane permeability by Bcl-2 proteins could be an important molecular mechanism of ER stress-induced apoptosis [30]. It has been shown that an ethanolic red propolis extract induces MCF-7 cell apoptosis mediated by ER stress-related signaling [13]. As shown here, BRP treatment increased the mRNA levels of the antioxidant enzymes CAT, Cu/Zn-SOD, TRx, GST, and Mn-SOD in a bladder cancer cell line. We have shown previously that hydroalcoholic extract obtained from red propolis presented high polyphenol content, important DPPH scavenging ability, and SOD-like and CAT-like activities [14]. Although further work needs to be carried out, the increased levels of the antioxidant enzymes observed in the present study might reflect the response of cells towards programmed death mediated by ER stress-related signaling.

In conclusion, our findings indicate that Brazilian red propolis induces cytotoxicity on superficial bladder cancer cells* in vitro* and this effect may be due to a caspase-independent apoptosis or apoptosis-like PCD. Additionally, these results are insightful for the antitumor effect of BRP and we speculate that red propolis may represent a source of therapeutic agents for bladder cancer.

## Figures and Tables

**Table 1:** Primers used for qRT-PCR in this study.

Gene	Sequence 5′-3′
p53 For	AGCGAGCACTGCCCAACA
p53 Rev	CACGCCCACGGATCTGAA
Caspase-9 For	CCAGAGATTCGCAAACCAGAGG
Caspase-9 Rev	GAGCACCGACATCACCAAATCC
Bcl-2 For	GTGTGGAGAGCGTCAACC
Bcl-2 Rev	CTTCAGAGACAGCCAGGAG
Endo G For	GTACCAGGTCATCGGCAAGAA
Endo G Rev	CGTAGGTGCGGAGCTCAATT
Bax For	ATGCGTCCACCAAGAAGC
Bax Rev	ACGGCGGCAATCATCCTC
Caspase-3 For	CAGTGGAGGCCGACTTCTTG
Caspase-3 Rev	TGGCACAAAGCGACTGGAT
Caspase-8 For	GGATGGCCACTGTGAATAACTG
Caspase-8 Rev	TCGAGGACATCGCTCTCTCA
GAPDH For	GGATTTGGTCGTATTGGG
AIF For	GGGAGGACTACGGCAAAGGT
AIF Rev	CTTCCTTGCTATTGGCATTCG
CuZn-SOD For	AGGGCATCATCAATTTCGAG
Cuzn-SOD Rev	TGCCTCTCTTCATCCTTTGG
Mn-SOD For	GGAAGCCATCAAACGTGACT
Mn-SOD Rev	CTGATTTGGACAAGCAGCAA
CAT For	TTTCCCAGGAAGATCCTGAC
CAT Rev	ACCTTGGTGAGATCGAATGG
GLUT For	TTCCCGTGCAACCAGTTTG
GLUT Rev	TTCACCTCGCACTTCTCGAA
GST For	CCCGATGTATCACGCAGTTA
GST Rev	TTCACTGCAACAGCAAAACC
TRX For	CTTGTGGCCTTTCTGAGGAG
TRX Rev	CTCTTGACGGAATCGTCCAT
GAPDH Rev	TCGCTCCTGGAAGATGG
